# Excision of a Scapula

**Published:** 1870-08

**Authors:** 


Excision of a Scapula.—Dr. Schuppert has excised the entire scapula of a woman 36 years old, for a tumor involving nearly the entire bone. The operation was recovered from promptly, the patient having a tolerably useful arm—one more useful, indeed, than before the operation. There was very little hemorrhage, partly due to the obliteration of some large vessels in a former operation. This is the tenth reported case of this operation.—*New Orleans Jour. Med.*

